# "Does replication groups scoring reduce false positive rate in SNP interaction discovery?: Response"

**DOI:** 10.1186/1471-2164-11-403

**Published:** 2010-06-24

**Authors:** Javier Gayán, Antonio González-Pérez, Agustín Ruiz

**Affiliations:** 1 Neocodex, Avda. Charles Darwin 6, 41092 Sevilla, Spain

## Abstract

A response to Toplak et al: Does replication groups scoring reduce false positive rate in SNP interaction discovery? BMC Genomics 2010, 11:58.

**Background:**

The genomewide evaluation of genetic epistasis is a computationally demanding task, and a current challenge in Genetics. HFCC (Hypothesis-Free Clinical Cloning) is one of the methods that have been suggested for genomewide epistasis analysis. In order to perform an exhaustive search of epistasis, HFCC has implemented several tools and data filters, such as the use of multiple replication groups, and direction of effect and control filters. A recent article has claimed that the use of multiple replication groups (as implemented in HFCC) does not reduce the false positive rate, and we hereby try to clarify these issues.

**Results/Discussion:**

HFCC uses, as an analysis strategy, the possibility of replicating findings in multiple replication groups, in order to select a liberal subset of preliminary results that are above a statistical criterion and consistent in direction of effect. We show that the use of replication groups and the direction filter reduces the false positive rate of a study, although at the expense of lowering the overall power of the study. A post-hoc analysis of these selected signals in the combined sample could then be performed to select the most promising results.

**Conclusion:**

Replication of results in independent samples is generally used in scientific studies to establish credibility in a finding. Nonetheless, the combined analysis of several datasets is known to be a preferable and more powerful strategy for the selection of top signals. HFCC is a flexible and complete analysis tool, and one of its analysis options combines these two strategies: A preliminary multiple replication group analysis to eliminate inconsistent false positive results, and a post-hoc combined-group analysis to select the top signals.

## Background

Epistasis, the interaction among genetic loci, is a frequent phenomenon in nature [1]. However, the detection of epistatic effects in observational data has not been an easy task because of the lack of appropriate samples and methodologies [2,3]. Thanks to the recent collection of large genetic datasets, we are now at a position where the study of epistasis in humans is becoming possible. Nonetheless, the genomewide evaluation of genetic epistasis is a computationally and statistically demanding task, due to the large number of possible combinations of loci that can be formed. For example, for a genomewide analysis with 100,000 SNPs, there are 5 × 10^9 ^two-locus combinations, and 1.7 × 10^14 ^three-locus combinations. For 1 million SNPs, there are 5 × 10^11 ^two-locus and 1.7 × 10^17 ^three-locus combinations.

The exhaustive search for epistasis across this large data space is a challenge for today's genehunters. In this context, a variety of software has been released to tackle this issue ([4] for review). HFCC (Hypothesis-Free Clinical Cloning) [4] is one of these tools that have made possible genomewide epistasis analysis. This software uses case-control samples to test for single-locus or multi-locus genetic association. Multi-locus combinations that are significantly associated with a trait are then subjected to a variety of post-hoc tests to determine the degree of non-additivity of the marker combination, that is, to separate additive multi-marker combinations from more epistatic interactions. Those genetic effects that are due to epistatic interactions are one of the priorities in our analysis, because they complement those effects detectable by single-locus analysis.

Because HFCC performs an exhaustive search of the entire data space, several optional tools have been implemented to overcome this multiple testing problem, such as multiple replication groups, the direction filter, the control filter, the tracking filter, etc. For example, the case-control sample can be simultaneously analyzed in replication groups, to select only significant results in each group. There is also a complementary direction filter, which selects only those results which are consistent across groups, that is, they are significant and with the same direction of effect in each group.

A recent article by Toplak et al. [5] has been inspired by the following statement in HFCC's article [[4], page 3]: "... a multi-group analysis strategy ... allows the replication of consistent results, and it also aids the elimination of false positive results, a very attractive quality for genome-wide analysis of large number of genetic markers." These authors have interpreted the above statement as claiming that using replication groups, by itself, reduces "the false positive rate" [[5], page 2], and can therefore "... improve ... any type of feature ranking and selection procedure ..." [[5], page 1].

Our approach to detecting multi-locus effects uses a two-stage analysis strategy. In a first step, a large subset of preliminary results that are associated with the disease are selected. Then, this liberal subset of results is subjected to a post-hoc analysis to select the most promising results [[4], page 7]. Using replication groups is only one of the possible analysis strategies of HFCC, aiming to reduce the number of selected signals, that is, it eliminates a larger amount of the tail of the distribution of the results, which are mostly false positives, together with some true effects that are undistinguishable from unassociated variants [10].

Our original statement claims that using multiple replication groups should reduce the number of signals selected with a liberal statistical threshold (mostly false positives), but does not claim to use this strategy to select the top results of the study. Indeed, to select the top findings, we analyze the combined sample [[4], page 6], which, as we state repeatedly across our article [[4], pages 2,3,4], is known to be the most powerful analysis strategy [6,7].

Therefore it seems Toplak et al. interpreted our article incorrectly, and applied this misinterpretation to test their own hypothesis (replication groups aids prioritization of signals), which was finally rejected by their simulations. In their paper, these authors provide evidence that the analysis of a combined sample is less prone to false positives than the separate analysis of replication samples. However, it is not clear from their article whether they have selected signals consistent in strength and direction, as suggested in our paper and in the guidelines for replication of association results [8], what may compromise to some extent their results. In this study, we have carried out HFCC analysis of several simulated datasets to evaluate the power and Type I error rate of the combined-group and replication-groups analysis strategies.

## Methods

We simulated several case-control datasets using a freely available software, genomeSIMLA [9]. Each dataset consists of 3000 cases and 3000 controls genotyped for 20 SNPs, with minor allele frequencies between 0.1 and 0.5. Different effect sizes were simulated, and each experimental dataset was simulated 100 times, so that we could estimate the power and Type I error rate under the defined parameters. The first experiment was a null simulation, where all 20 snps were simulated under the null hypothesis of no association between the SNPs and case status. Next, we performed 3 experiments, each with 3000 cases and 3000 controls, 18 null SNPs, and one pair of SNPs in epistatic association with case-status under different effect sizes. The SNP pair was designed to have a MAF = 0.3 for each SNP, having a larger penetrance (.08) for only one genotype, the double-homozygote (AAbb). A background penetrance of 0.05 (for all other genotypes) versus the AAbb penetrance of 0.08 resulted in a mean Odds-Ratio (OR) of 1.67 (Range = 1.34-2.25). Decreasing the background penetrance to 0.04, resulted in a mean OR of 2.10 (Range = 1.64-2.75). Finally, a background penetrance of 0.03 resulted in a mean OR of 2.85 (Range = 1.94-3.87).

In addition to these experiments, a mixed simulation was carried out, in wich 3 different case-control samples (ie, replication groups) were simulated, each with 1000 cases, 1000 controls, and 20 SNPs. Two replication groups were simulated under the null hypothesis of no association. A third replication group was simulated with 18 null SNPs, and a pair of SNPs in epistatic association with background penetrance of 0.03 and AAbb penetrance of 0.08. These 3 replication groups were then merged for a combined group analysis with an average OR of 1.57. This experiment simulates an scenario to search for consistent association results, where one case-control sample shows an association which could be real or could be due to sampling or genotyping artifacts, and two other samples which do not exhibit this association.

## Results

We analyzed each simulated dataset with the HFCC software, using two-strategies: A combined-group analysis with a significance level of 10^-3^, and a three-replication-group analysis, with a significance level of 10^-1 ^for each group. This second strategy was applied with and without the direction filter (consistency of direction of effect across groups). HFCC was employed to test for association between all two-locus SNP combinations and case-status (20 SNPs produce 190 two-locus combinations). For each bi-SNP combination, nine simple genetic models were tested, where each test compares the frequency of one of the nine possible two-locus genotypes (AABB, AABb, AAbb, AaBB, AaBb, Aabb, aaBB, aaBb, and aabb) against the frequency of the remaining genotype classes.

The null simulations resulted in a Type I error rate of 0.00080 for the combined analysis, and 0.00086 for the 3-group analysis. The observed type I error rate is a bit conservative compared to the theoretical rate (0.001), which is probably due to the fact that some of the genetic model tests are correlated. In any case, both analysis strategies are similar and conservative. Most importantly, when the direction filter is applied, the Type I error rate drops to 0.0002, much lower than for the two previous analyses, proving that the replication group analysis, when the direction filter is applied, reduces the false positive rate.

We then analyzed three datasets simulated under three different effect sizes (Average OR of 1.67, 2.10, and 2.85). Table 1 summarizes these results, where we find that the combined analysis is, as expected and suggested in our original paper [4], always more powerful than the 3-group analyses. Nonetheless, and regarding Toplak et al.'s main claim [5], the false positive rate decreases when we use the 3-group strategy, and more so when we apply the direction filter.

**Table 1:** Power and Type I error rates for several simulated datasets and analysis strategies.

		OR			
		
Analysis Type		1.67	2.10	2.85	1.57 Mixed
**Combined group (p = 10**^**-3**^**)**	Power	90	100	100	71
	False positive rate	0.00379	0.01144	0.02448	0.00297
**3 group No Direction (p = 10**^**-1**^**)**	Power	57	100	100	0
	False positive rate	0.00197	0.00538	0.01713	0.00108
**3 group Direction (p = 10**^**-1**^**)**	Power	57	100	100	0
	False positive rate	0.00121	0.00486	0.01645	0.00039
**Combined group (p = 10**^**-4**^**)**	Power	66	100	100	50
	False positive rate	0.00103	0.00571	0.01824	0.00070

Each dataset consists of 3000 cases and 3000 controls genotyped for 20 SNPs, and each experimental dataset was simulated 100 times. The simulated effect size for each experiment (a pair of SNPs per simulation) had an average Odds-Ratio (OR) of 1.67, 2.10, and 2.85. A final mixed model (overall OR of 1.57) resulted from the combination of two null simulations with a simulation with a pair of SNPs associated with case-status. The combined-group strategy analyzed the full sample size simultaneously (3000 cases and 3000 controls), and was applied for two different significance levels (p = 10^-3 ^and p = 10^-4^). The 3-replication-group strategy analyzed three replication groups, each with 1000 cases and 1000 controls, with a significance level of p = 10^-1 ^in each group. Results with and without applying a direction filter (consistency of results in size and direction of effect across replication groups) are shown. Power is expressed as a percentage.

It has also been suggested [5] that the false positive rate can be reduced by increasing the significance level in a combined group analysis. The bottom row of Table 1 shows that a combined analysis using a significance level of 10^-4 ^achieves a significance level similar than the 3-group strategy, and a greater power. But, of course, this is not a fair comparison: If we apply this new significance level with a 3-group strategy, we would obtain again a reduction in the false positive rate.

A final comparison is reported in the last column of Table 1. We simulated a mixed population, where one replication group produces an association signal, while two other replication samples are simulated under the null hypothesis of no association. We found that the combined group analysis detected the association 71 and 50 percent of the time, but the 3-group analysis did not detect it even once. In addition, the false positive rate was lowest for the 3-group analysis with direction filter.

## Discussion

We have evaluated the power and Type I error rate of two possible analysis strategies: a combined group analysis of the full sample, and a replication group analysis. The results presented in Table 1 and in the Results section show that applying a replication group analysis with a direction filter reduces the false positive rate relative to a combined group analysis. It is important to note here that the combined analysis is more powerful, something already mentioned in our original paper, and therefore is preferable for selecting the top signals of a study. Nonetheless, when lack of replicability of results is an issue, using a multiple-group strategy is an useful tool to select a large subset of results that are consistent across groups, and that may replicate in future independent replication studies.

As we stated in our original article [4], the replication groups strategy is used to select a large subset of preliminary results, which are then subjected to post-hoc analysis for prioritization. We used the replication groups to eliminate the tail of false positive results, not to choose the top best signals (scoring or ranking), which is done in a post-hoc analysis. This is a key misinterpretation of our strategy in the Toplak et al. article, and, although we will not mention them all here, there are several others. For example, they claim we did not compare in our original publication a multiple-group strategy versus a combined group strategy [[5], page 1], but we compared them and stated that the analysis of the Parkinson Disease dataset, in a single combined group, yielded 784,506 pairs of SNPs at a p-value of 10^-6^. The same analysis, with 3 replication groups each at p-value of 10^-2^, yielded only 418,535 pairs [[4], page 12]. Moreover, when the direction filter was applied to this 3-group strategy, then the analysis only yielded 320,265 pairs [[4], Table 2], a significant decrease.

When the multiple group strategy is used, it is important to use the direction filter, to select only those signals significant in each group but also consistent in the direction of effect. Our original simulations suggested this filter may eliminate about 24% of the signals selected by the 3-group analysis [[4], pages 7-8]. It is not clear from their article whether Toplak et al. have applied a direction filter. It seems they have selected signals above a statistical threshold, but may have failed to select only those results with the same direction of effect in all groups. If this is true, then the results published in their article [5] are compromised, because it seems straightforward that selecting only consistent results, in both strength and direction, will reduce the number of false positives selected in the multiple-group analysis. Indeed, the results presented in Table 1 and in the Results section show that applying a replication group analysis with a direction filter lowers the false positive rate relative to a combined group analysis.

Toplak et al. also argue that instead of using multiple replication groups, a decrease in false positives can be achieved by simply raising the significance level in a combined group analysis [[5], page 7], a statement with which we obviously agree. However, if this new significance level is applied with multiple replication groups, the false positive rate would be lower again than for the combined analysis.

We have also shown that when the replication groups are heterogeneous, a combined group analysis may detect the signal that is due to a subset of the sample, but a multiple-group analysis with direction filter protects against this potential source of bias. Obviously, if there is heterogeneity across samples and the effect is real only in some subsets, the combined analysis is more powerful to detect a potentially true effect, even though the effect could be erroneously generalised to all three groups. However, if the goal is to find results consistent across replication groups, and protect against false positive results, then the replication-group strategy provides this added value.

We also want to note that our multiple group strategy has its roots in the ability to analyze simultaneously multiple related phenotypes (such as comorbid or related diseases). This is another advantage of HFCC, which is inherently designed to allow this type of analysis. Moreover, some gene factors may have a risk effect on a disease, and a protective effect on another one, a possibility that can only be addressed with multiple-groups and flexible direction filters.

## Conclusion

Replication of results in independent samples is generally used in scientific studies to establish credibility in a finding [8]. It protects against bias from unmeasured sources of noise (stratification, sampling or selection bias, technical artefacts or non-random genotype errors, heterogeneity across samples). Replication across samples has become the standard strategy in recent years with the proliferation of genome-wide studies of many diseases. Nonetheless, the combined analysis of several datasets is known to be a preferable and more powerful strategy [6,7] for the selection of top signals. HFCC [4] is a flexible analysis tool, and one of its analysis options combines these two strategies: A preliminary multiple replication group analysis to eliminate inconsistent false positive results, and a post-hoc combined-group analysis to select the top signals. HFCC, however, is a more complete software which includes other analysis options and methodologies. This article explains these concepts and shows that using replication groups with direction of effect lowers the false positive rate.

As a summary of our results, if the goal of an analysis is to select the best top signals, a combined group analysis provides the most powerful approach. Subdividing a large homogeneous sample into several smaller subsets is not generally recommended in this case, unless there is some evidence of heterogeneity. When there exist several independent and heterogeneous samples, different in case selection, geographical location, race, genotyping, etc., or even sample size, applying a multiple replication group strategy may help eliminate false positive signals and select results consistent across groups. Genomewide studies of large datasets are showing that, for reasons not clear yet, but probably due to sampling, technical or genotyping differences, the top signals are not consistently replicating in independent samples (the winner's curse). On the contrary, signals down the top rankings are the ones finally replicating. Because in the recent history of genetic studies of complex traits it has been hard to find consistent results [8], and the level of noise to true signals is large [10], a replication group strategy seems useful for this sort of studies.

## List of abbreviations

HFCC: hypothesis free clinical cloning; MAF: minor allele frequency; OR: Odds-ratio.

## Competing interests

All authors in the paper are employees and/or shareholders in Neocodex. Neocodex owns a patent on the HFCC algorithm described in this paper.

## Authors' contributions

JG wrote the paper. JG, AGP and AR have discussed the topic, read, revised and approved the final manuscript.
